# Cultural persistence of self-assessed health: A study of first- and second-generation migrants

**DOI:** 10.1016/j.jmh.2024.100280

**Published:** 2024-10-28

**Authors:** Joan Costa-Font, Azusa Sato, Belen Saenz-de-Miera

**Affiliations:** a London School of Economics and Political Science (LSE); b Autonomous University of Baja California Sur (UABCS), Mexico

**Keywords:** Wellbeing, Mental health, Public health, Global health, Health care, Healthcare, Health issues, Mental wellness, Cultural persistence, Culture

## Abstract

**Objectives:**

We measure the cultural persistence of health assessments; namely the association between first (and second) generation migrants' health assessments and those of their home country (and that of their parents).

**Measure:**

We use individual data records from over thirty host European countries and over ninety sending countries, as well as controls for migration timing and legal citizenship status. Furthermore, we leverage a wide range of sample countries to attenuate the presence of selection bias.

**Results:**

Our estimates document evidence of cultural persistence of health self-assessments in a wide array of different specifications which vary with age. We estimate that a one standard deviation change in self-reported health in the sendning country is associated with an increase in migrants’ self-reported health of about 0.17 standard deviations. The effect size is sensitive to the inclusion of country of residence fixed effects as well as the presence of selection on observables and other robustness checks.

**Conclusion:**

Cross-country comparisons of self-reported health should consider cultural reference points individuals use in assessing their health.

## 1 Introduction

Health-related cultural norms serve as reference points that influence how individuals assess their health. These norms, however, are shaped by personal expectations and narratives, which play a key role in how people experience, interpret, and maintain their health (WHO 2008; Napier et al., 2017, Hernandez and Gibb, 2020). For instance, some evidence reveals that culturally specific practices can influence ‘objective health status’ by limiting the spread of diseases in epidemics or offering protection against communicable and non-communicable health threads (Hewlett, 2007). Although health assessments are well known to correlate with objective measures of health status, to date, there is limited empirical evidence of how culturally persistent such health self-assessments are, which limits their cross-country comparability.

An opportunity to study cultural influences is by examining samples of individuals who have migrated to countries from a specific sending country where we can measure self-assessed health too. This is the case because the effect of culture partially varies with some slow-moving features such as language and traditions (Hernandez and Gibb, 2020). Migrant samples allow studying the effect of cultural persistence once we control for citizenship regulations, welfare institutions, or the duration of an individual's residence in a country. The intuition behind the methodology is that health assessment priors can be conceived as portable reference points of what is regarded as ‘good’ or ‘bad’ health. Hence, a measure of the cultural persistence of health assessments can be extracted by examining the systematic association between migrants’ health assessments and the health assessments of individuals from their home countries. This is possible in surveys that contain large samples of immigrants from multiple sending and host countries to mitigate potential selection biases.

‘Cultural persistence’ refers to the paucity of the culture individuals are being brought up in, namely the extent to which health assessments of migrants are influenced by the culture of the sending countries. Accordingly, we primarily focus on evidence from second-generation migrants, who have grown up in their host country, but might still hold cultural priors aligned with the health assessments of their parents’ sending country’. This is true, insofar as the persistance of health assessments is not biased by the effect of the host institutions, and especially when additional controls are included for citizenship. However, migrants do not qualify as being part of a random sample of their population of residence, so in examining immigrant data it is especially important to control for any characteristics that make immigrants different from the rest of the population, including the fact that first-generation migrants have not been brought up in the host country while second generation migrants might have.

In this paper, we investigate the relationship between the health assessments of migrant individuals—whose parents or themselves were not born in the host country—and the average health assessments of their (or their parents') sending (or home) country. We draw upon seven waves of the European Social Survey (ESS) 2004 - 2016 containing self-reported health records from 30 different European member states. The ESS is unique in that it contains a consistent measure of self-assessed health and allows us to include several controls for important alternative explanations that could drive the association between migrants and their home countries' health assessments. Such controls can help identify some of the potential sources of migrant selection (for example, time in the host country or citizenship), as migrants may differ from population averages in key observable dimensions.

Nonetheless, an important methodological concern when using migrant records is that the health status of immigrants at the time of migration might be better than that of natives. Given that migration is not a random process, but a rather costly one, only those individuals who are healthy enough to bear the associated costs might undertake the move (also know as the ‘heathy migrant’ effect). However, evidence from European migrants calls the 'healthy migrant effect' into question (Constant et al., 2018).

We make several contributions to the literature. First, we advance the discussion on the cultural determinants of health assessments, an area that has been underexplored thus far. Our findings also extend beyond the health assessments of first-generation immigrants (Salant and Lauderdale, 2003) and measures of happiness (Ljunge, 2016). Specifically, we provide evidence that culture exerts a long-term influence by shaping the reference points individuals use when assessing their own health. If we were to compare two individuals in the same health state but who assess their health differently, then this difference could be interpreted as stemming from different cultural reference points in the assessment of their own health status. Second, previous research has used individuals' health in their country of origin as an instrument to exploit the exogenous variation in health assessments, allowing for the examination of its impact on labor market decisions (Costa-Font and Ljunge, 2018), but it does not examine the cultural transmission mechanisms. This paper considers a number of potential threats, biases and potential genetic effects by adding objective measures of health. Finally, this research contributes to the so-called ‘epidemiological approach’ literature that compares immigrants' preferences to the average preferences of people in their countries of birth which has been used to explain the use of traditional medicines (Costa-Font and Sato, 2024), and differences in savings (Costa-Font et al., 2018). We study the cultural persistence of such assessments, how robust such persistence is to the inclusion of country of residence fixed effects and different subsamples. Next, we examine a number of mechanisms to understand different explanations for the cultural effect. Its worth mentionning that the paper most closely related to ours is Roudijk et al. (2017), which explores how country-of-origin influences health and well-being assessments. However, this literature does not address the cultural persistence or the mechanisms that underpin such health assessments.

We find evidence consistent with the presence of strong cultural persistence in health assessments. Our estimates are robust to a series of robustness checks, empirical strategies, and the addition of an important set of controls that account for different forms of selection. Finally, our estimates reveal heterogeneous effects by gender, age, and region.

The structure of the paper is as follows. The next section discusses previous research, sections two and three reports the data and the empirical strategy. Section four contains the results, followed by robustness checks, and the final section concludes.

## 2 Background

*Culture and health*. Culture refers to a system of shared understandings and values that can influence the reference points individuals use in making health assessments (Napier et al., 2017). Such shared values can act as triggers (or barriers) for certain behaviours, such as seeking health care, or spending time in or near natural landscapes (Napier et al., 2017). It is conceivable that cultural reference points exert a direct impact on how people perceive health, for instance influencing how illness and pain are perceived. More specifically, health professionals in some European countries such as Belgium, Switzerland, and Germany, employ the term “Mediterranean syndrome” to refer to individuals who “are known for their tendency to present with diffuse complaints and exaggerate pain” (Ernst, 2000).

*Healthy migrant effect (HME)*. Examining the health assessment of individuals transitioning from one culture to another can help identify the role of cultural reference points. Previous studies using migrants’ records have provided rich evidence of how migrants adapt to a new culture, and more specifically, how health outcomes are influenced by time spent in a country. Indeed, migrants are argued to exhibit ‘protective cultural factors’ such as a healthier lifestyles.

Nonetheless, the health advantage of migrants declines with time spent in the host country. For instance, the health of Latin American migrants to the United States appears to deteriorate as they stay in the country longer, indicating an unhealthy adaptation (Kaestner et al., 2009). However, other evidence suggests that the longer an immigrant stays in the country, the better their health (Jasso, 2003). Indeed, although some evidence suggests that health benefits are lost in childhood (Hamilton et al., 2011), and many health conditions worsen across generations, exposure to a new environment can trigger the adoption of native behaviors (Mendoza, 2009). Yet, such healthy migrant advantage disappears in European countries which might be explained by the fact that migrants come from a larger set of sending countries compared to the United States, and there is a large variation in host cultures (Napier et al., 2017). Constant et al. (2018) did not find evidence of a healthy migrant effect in Europe. Hence, Europe is an ideal setting to study the cultural persistence of health assessments, given its large variation in cultures and lesser exposure to migrant selection.

## 3 Data

### 3.1 The data

We draw upon data from the European Social Survey (ESS), and more specifically waves 2 to 8, measuring the health self-assessment of Europeans every two years between 2004 and 2016 inclusive (European Social Survey Cumulative Files, 2020).^1^ All cross-sections were first merged, and then variables made consistent across waves. The data includes 30 host countries, and the survey contains information about the respondents’ sending country or that of his/her father and mother.

Individual-level data from the ESS was matched with health assessment measures constructed at the country level from the World Values Survey (WVS) for over 90 countries (Inglehart, 2020). The World Values Survey contains data for many countries, but the survey is conducted every five years (wave 1: 1981–1984, wave 2: 1990–1994, wave 3: 1995–1998, wave 4: 1999–2004, wave 5: 2005–2009, and wave 6: 2010–2014), and samples of countries frequently exhibit significant attrition. As a result, the sample is used to compute average health assessments in the home country from 2000 to 2014, though lag averages from 1981 to 1998 are also used in robustness checks. We also account for per capita health expenditure in the country of residence, as retrived from the World Bank database.

For all waves, we use self-reported health assessments, which allows us to take advantage of variations in health assessments over time in host countries. However, health measures may be more dependent on changes in individual specific circumstances rather than changes in context (e.g., migration). We draw on two samples from our master dataset: one for first-generation migrants (people born in one country who moved to another) and one for second-generation migrants (defined as children of first-generation immigrants, e.g., those with one or both parents not born in the same country as the child). There were 24,880 and 22,319 observations in these analytic samples, respectively.

### 3.2 Dependent variables

Our primary variable of interest is self-reported health, which is assessed subjectively on a five-point scale ranging from very good to very bad. The question posed is: “How is your health in general?” Respondents can choose from the following options: very good, good, fair, bad, or very bad (Table A1 in the Appendix for details). It is important to acknowledge that while self-reported health is the most used measure of health, it is not without its biases and can show inflated responses and significant cross-country variation (Jürges, 2007). Given that health assessments are a proxy for latent health, cultural biases in self-assessments are a proxy for cultural effects on health. To analyze some of these effects, we carry out subsample analysis where the composition of the countries differs, alongside analysis of measures of health that are not directly self-reported.

### 3.3 Independent variables

Our key explanatory variable refers to the average health assessments in the sending country, specifically distinguishing both the father and mother's country of birth for second generation migrants. Given that the correlation of health assessments can be explained by other potential pathways, we include several controls. Such controls capture individual-specific conditions that can independently influence the way health is individually assessed. Furthermore, given that health declines with age and exhibits gender and household-specific differences, we control for several socioeconomic and demographic characteristics (gender, age, and household size). Institutional explanations for an association in migrant's health assessments such as citizenship status are also considered. These are important measures, as in some countries migrant's citizenship is not automatic after birth. Our data also contains records on how long individuals have lived in the country of residence, and whether they belong to a minority ethnic group. Alongside educational attainment, we include main occupational activity and household net income quintile, which measure socio-economic determinants of health. The baseline specification includes wave controls.

### 3.4 Migrants and health in Europe

In Europe, free mobility between member states ensures limited barriers to the access health care across countries of the European Union. Rights are more restricted to undocumented migrants, although they have a right to health care under legal conventions of the European Union as established in article 35 of the EU Charter of Fundamental Rights. However, countries can differ in whether they provide care beyond emergency care in the first instance. Hence, in our analysis we will perform a specific heterogeneity analysis distinguishing the origin of migrants to account for differences in their rights.

## 4 Empirical strategy

### 4.1 Data considerations

Summary statistics are reported in Table A1 in the Appendix. Consistent with studies using the same data, we find that immigrants compare to the general population on many observable variables, with some differences in religion and education, which we control for along with several other controls. In our analysis we specifically distinguish first-generation (migrants themselves) and second-generation migrants (children of migrants).

We assume that first-generation migrants have been affected by the institutions of both home and host countries and might even have been affected by transition costs. Hence, the results for first-generation migrants do not reflect cultural effects alone but are influenced by other effects that we capture when we examine the effect of time in the host country. Similarly, given that first-generation migrants chose to migrate themselves, one can expect first-generation migrants to have more incentives to adopt the health-related norms of the destination country, and hence there might be some selection into migration to certain countries based on, for instance, attitudes of the host population.

In contrast, second-generation migrants have been raised in the same country as natives and did not choose their country of birth. Hence, if controls for alternative mechanisms are included, evidence of correlation in health assessments suggests cultural persistence in health assessments.

### 4.2 Model specification

Based on the above considerations, we examine the association between migrants’ health assessments and that of their sending country using a reduced form estimate that draws on the following specification:

(1) $$H_{ijt}=\;\rho {\bar{H}}_{j}+\;\varphi X_{it}+\mu_{t}+\varepsilon_{ijt}$$

where $H_{ijt}$ is self-reported health of first (second) generation migrant i from the sending country j at time t, ${\bar{H}}_{j}$ refers to the sending country health assessment for either first or second-generation migrants retrieved from the World Value Survey, $X_{it}$ refers to individual-specific controls that could bias our estimates of cultural persistence, and $\mu_{t}$ are fixed wave effects. Our coefficient of interest is ρ, measuring the association between the migrant's health assessment and the average health assessment in the sending country. $\varepsilon_{ij}$ indicates random parameter, which may include country-of-residence fixed effects. Country of origin fixed effects are not included in this literature as they absorb the entire effects of cultural norms and values influencing country health assessments.

To account for the arbitrary correlation of error terms among individuals from the same country of origin, standard errors are clustered at the individual's country of origin. For robustness purposes, we estimate both linear probability models and ordered probit models. The results are presented in standardised coefficients to compare the mean between the first and second generations; marginal effects for nonlinear models are also included.

### 4.3 Heterogeneous effects and robustness checks

We run several specifications in addition to our baseline models to investigate heterogeneous effects and address potential biases. We focus on specifications that distinguish between paternal and maternal lineage for second-generation migrants. This is important when second-generation migrants come from different countries, hence we can distinguish the influence of the maternal and paternal country of origin. We consider cohort differences, as early life health assessments may reflect differences in reference points for what constitutes "good health" when compared to other categories, whereas later life health assessments may reflect true differences in health status. Heterogeneous effects by gender and region are also analysed. Other estimates include regional and country of residence fixed effects to account for any unobserved time-invariant characteristics, lags in average health assessments of the home country as migrants might not observe contemporaneous values when making their judgements, and other measures of wellbeing. In addition, we define cohorts based on gender and year of birth and restrict our analysis to migrants from European countries who have similar rights in both host and sending country. Given that mobility restrictions within Europe are less stringent for European citizens, the analysis of this subsample of migrants allows examining potential sources of unobserved heterogeneity that could not be entirely controlled for with destination country fixed effects.

## 5 Results

### 5.1 Descriptive evidence

**Fig. 1:**
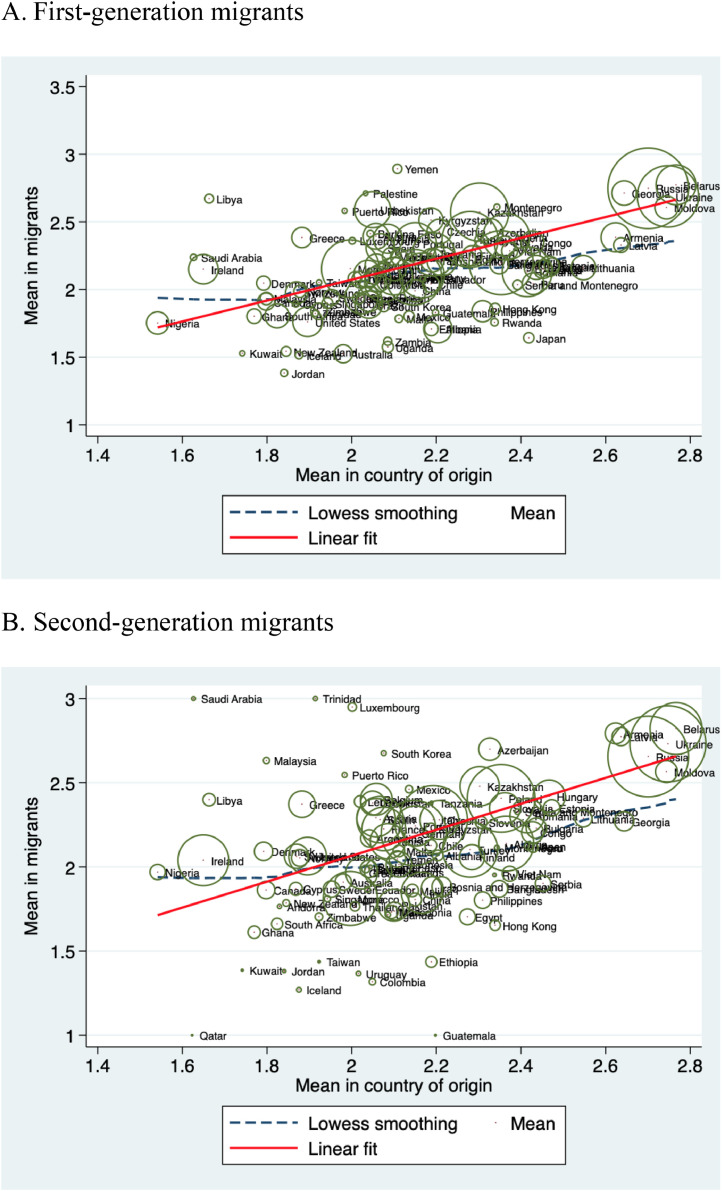
Cultural persistence of health capital. Correlation of self-assessed health between country of origin and first- and second-generation migrants. Note: The size of the circles represents the number of migrants from each country.

Fig. 1 shows the association between the self-assessed health of first- and second-generation migrants and the average health capital in their country of origin. The size of the circles depicts the number of migrants from each country. Estimates show the fitted values of the association between the two measures. Indeed, for both first- and second-generation migrants, the fitted values indicate a steep and positive association consistent with the presence of some cultural persistence in health assessments.

Table A2 in the Appendix displays more detail on self-reported health patterns among first- and second-generation migrants and natives. In general, second-generation migrants have better average self-reported health. We also observe the typical gradients in self-reported health by age, education, employment status, and income groups for all first- and second-generation migrants and natives, although in some cases the differences between extreme categories are greater for first-generation migrants (e.g., for age groups) or natives (e.g., for education and income groups).

### 5.2 Baseline estimates

*Cultural Persistence:* In Panel A of Table 1, we report the regression estimates for first-generation migrants only. We examine estimates both without and with controls (columns 1–2 and 3–4, respectively), using inear and nonlinear models. Given that migrants’ behaviors might change with exposure to the host country, we then include citizenship status and time in the country since arrival (columns 5–6). More specifically, we specify five dummy variables: whether individuals have spent less than 1 year in the country of residence (reference), between 1 and 5 years, between 6 and 10 years, between 11 and 20 years, and more than 20 years. In all cases, the estimates suggest a large and significant coefficient of health assessments of the migrants’ home country consistent with the hypothesis of cultural persistence. As expected, the size of the cultural persistence coefficient declines with the inclusion of socio-economic and demographic controls. We find that spending up to ten years in the host country increases cultural attachment to the country of origin, and the coefficient is even larger when migrants have been in the country of residence for more than 20 years.

**Table 1:** Cultural persistence of health status. Baseline models.

		OLS	Oprobit	OLS	Oprobit	OLS	Oprobit
		(1)	(2)	(3)	(4)	(5)	(6)
Panel A. First-generation migrants							
Self-assessed health at country of origin		0.880***	0.974***	0.600***	0.774***	0.584***	0.757***
		[0.248]	[0.048]	[0.169]	[0.033]	[0.165]	[0.031]
		(0.150)	(0.169)	(0.060)	(0.084)	(0.058)	(0.081)
Citizen of country of residence						0.029	0.038
						(0.025)	(0.032)
Time in country of residence							
Within last year (reference)							
1 to 5 years						0.036	0.083
						(0.052)	(0.083)
6 to 10 years						0.092*	0.167*
						(0.055)	(0.086)
11 to 20 years						0.153**	0.253***
						(0.063)	(0.096)
More than 20 years						0.280***	0.417***
						(0.067)	(0.102)
Observations		24,880	24,880	24,880	24,880	24,457	24,457
R^2^ / Pseudo R^2^		0.06	0.02	0.29	0.12	0.29	0.12

Panel B. Second-generation migrants							
Self-assessed health at country of origin	0.758***		0.892***	0.573***	0.766***		
	[0.225]		[0.031]	[0.170]	[0.024]		
	(0.083)		(0.106)	(0.055)	(0.079)		
Observations	22,319		22,319	22,319	22,319		
R^2^ / Pseudo R^2^	0.05		0.02	0.25	0.11		
Wave fixed effects	Yes		Yes	Yes	Yes	Yes	Yes
Controls	No		No	Yes	Yes	Yes	Yes

Notes: The dependent variable is self-assessed health of first- and second-generation migrants who live in European countries (SAH=1 very good,…, SAH=5 very bad). Standardised coefficients (OLS models) and average marginal effects on the probability of the worst self-assessed health (Oprobit models) are in brackets. Standard errors (in parenthesis) are clustered at the country-of-origin level. Specifications with controls (columns 3–6) include gender, age, education, marital status, household size, religion, whether belongs to minority ethnic group, employment status, and household income (quantiles). * *p* < 0.1; ** *p* < 0.05; *** *p* < 0.01.

Table A4 in the Appendix includes additional controls, namely regional fixed effects, country-of-residence fixed effects, and per capita health expenditure in the host country (Panel A). The specification with fixed effects for five regions of Europe (North, South, Centre, East, and West) yields similar results (columns 1–2), although the more specific country-of-residence fixed effects, which capture time-invariant differences between countries, do reduce the size of the coefficient of interest (columns 3–4). Something similar is observed when the per capita health expenditure in the country of residence is considered (columns 5–6).

Next, we include lagged values of average health in the country of origin to rule out the possibility that some unobserved variables are simultaneously affecting the health status of immigrants and natives (e.g., international epidemics; columns 1–2 of Table A5 in the Appendix). Estimates are comparable to those in panel A of Table 1. Finally, panel A of Table A6 in the Appendix adds survey weights to the estimates, revealing no differences from those in Table 1.

*Cultural Effects: Second Generation.* Results for the first generation cannot be interpreted as evidence of cultural effects alone as migrants might not be subject to the same regulations as natives. Hence, panel B of Table 1 reports the same estimates but for second-generation migrants (e.g., children of migrants) who have been raised in the same institutional environment as natives. Consistent with the results for first-generation migrants in panel A of Table 1, we report estimates without and with controls (columns 1–2 and 3–4, respectively) as before. Cultural persistence for second-generation migrants is practically the same, as descriptive evidence already suggests. More specifically we estimate that a one standard deviation change in the country of origin self-reported health is associated with an increase in migrants’ self-reported health of about 15–17 standard deviations. Only in the specification with country of residence fixed effects (columns 3 and 4 in Panel B of Table A4 in the Appendix), the coefficient of interest is less sensitive to considering an ordered probit specification rather than linear probability estimates.

Table A7 in the Appendix shows the results using an alternative definition of second-generation migrants that distinguishes whether the father (columns 1–3) or mother (columns 4–6) was born abroad. Importantly, we find that cultural persistence is only slightly higher for second-generation migrants when measured along paternal lineage, but the difference between the two coefficients is not statistically significant.

### 5.3 Heterogeneous effects

*Gender Effects*. In Table 2 we report the results for both first- and second-generation migrants (Panel A and B, respectively), splitting the sample by gender. Consistently, we find significant and large coefficients that do not differ considerably by gender. A change in one standard deviation in the country-of-origin's self-assessed health increases migrants’ self-assessed health by nearly 0.60 scale units (16%) irrespective of gender (columns 1 and 2). Table A8 in the Appendix again distinguishes paternal and maternal lineage (panels A and B, respectively). The effect decreases to 0.50 scale units (15 % compared to the mean) on maternal lineage among men. However, among women, the effect is virtually the same for second-generation migrants of both maternal and paternal lineage.

**Table 2:** Cultural persistence of health status. Heterogeneous effects by gender.

	OLS		Oprobit	
	Female	Male	Female	Male
	(1)	(2)	(3)	(4)
Panel A. First-generation migrants				
Self-assessed health at country of origin	0.608***	0.578***	0.774***	0.763***
	[0.172]	[0.162]	[0.037]	[0.027]
	(0.066)	(0.059)	(0.092)	(0.084)
Wave fixed effects	Yes	Yes	Yes	Yes
Controls	Yes	Yes	Yes	Yes
R^2^ / Pseudo R^2^	0.30	0.27	0.12	0.11
Observations	13,822	11,058	13,822	11,058

Panel B. Second-generation migrants				
Self-assessed health at country of origin	0.587***	0.556***	0.773***	0.760***
	[0.174]	[0.166]	[0.025]	[0.023]
	(0.064)	(0.053)	(0.095)	(0.074)
Wave fixed effects	Yes	Yes	Yes	Yes
Controls	Yes	Yes	Yes	Yes
R^2^ / Pseudo R^2^	0.26	0.23	0.11	0.10
Observations	12,062	10,257	12,062	10,257

Notes: The dependent variable is self-assessed health of first- and second-generation migrants who live in European countries (SAH=1 very good,…, SAH=5 very bad). Standardised coefficients (OLS models) and average marginal effects on the probability of the worst self-assessed health (Oprobit models) are in brackets. Standard errors (in parenthesis) are clustered at the country-of-origin level. Controls include gender, age, education, marital status, household size, religion, whether belongs to minority ethnic group, employment status, and household income (quantiles). * *p* < 0.1; ** *p* < 0.05; *** *p* < 0.01.

*Age and Geographical Effects*. Next, we explore other specifications to try to disentangle whether our estimates could be partly attributed to genetic transmission rather than cultural transmission (Table 3). Specifically, we split the sample by age group (panels A and B for first- and second-generation migrants, respectively) and region of Europe (panels C and D for first- and second-generation migrants, respectively). In the first case, we find statistically significant effects for all age groups that roughly correspond to age quartiles (35 years or less, 36 to 50 years, 51 to 65 years, 66 years, and more), although we find a very clear positive gradient. These results suggests that, even among younger age groups where individuals typically exhibit very good self-assessed health, we still find consistent evidence of cultural transmission, implying that that there are relevant differences in cultural reference points when making health self-assessments across individuals. Significant results, on the other hand, are found for five regions based on country of residence (North, South, Center, East, and West), though with significant variations. The coefficients for first-generation migrants, for example, are estimated to range from 0.169 in the South to 0.673 in the North. When we look at second-generation migrants, however, we find no evidence of cultural transmission in the Southern and Eastern countries. That is, cultural persistence is primarily driven by cultural persistence in Northern and Central European countries.^2^

**Table 3:** Cultural persistence of health status. Heterogeneous effects by age group and regions of Europe.

	(1)	(2)	(3)	(4)	(5)
AGE GROUP	35 years or less	36–50 years	51–65 years	66+ years	
Panel A. First-generation migrants					
Self-assessed health at country of origin	0.239***	0.613***	0.707***	0.927***	
	[0.081]	[0.192]	[0.217]	[0.270]	
	(0.051)	(0.072)	(0.060)	(0.118)	
Wave fixed effects and controls	Yes	Yes	Yes	Yes	
R^2^	0.04	0.11	0.19	0.20	
Observations	6677	6965	5914	5324	

Panel B. Second-generation migrants					
Self-assessed health at country of origin	0.444***	0.544***	0.649***	0.710***	
	[0.156]	[0.172]	[0.196]	[0.206]	
	(0.060)	(0.067)	(0.078)	(0.095)	
Wave fixed effects and controls	Yes	Yes	Yes	Yes	
R^2^	0.09	0.18	0.18	0.14	
Observations	7666	6136	5346	3171	
**Regions of Europe**	**North**	**South**	**Center**	**East**	**West**

Panel C. First-generation migrants					
Self-assessed health at country of origin	0.673***	0.169**	0.508***	0.376***	0.248***
	[0.221]	[0.045]	[0.104]	[0.107]	[0.049]
	(0.101)	(0.075)	(0.091)	(0.107)	(0.086)
Wave fixed effects and controls	Yes	Yes	Yes	Yes	Yes
R^2^	0.35	0.17	0.22	0.37	0.16
Observations	8093	2187	6517	5174	2843

Panel D. Second-generation migrants					
Self-assessed health at country of origin	0.625***	−0.062	0.369***	0.147	0.266**
	[0.253]	[−0.012]	[0.072]	[0.043]	[0.042]
	(0.081)	(0.112)	(0.084)	(0.090)	(0.108)
Wave fixed effects and controls	Yes	Yes	Yes	Yes	Yes
R^2^	0.24	0.30	0.24	0.38	0.15
Observations	6459	760	6342	5559	3156

Notes: The dependent variable is self-assessed health of first and second generation migrants who live in European countries (SAH=1 very good,…, SAH=5 very bad). OLS estimates, standardised coefficients are in brackets; standard errors (in parenthesis) are clustered at the country of origin level. Controls include gender, age, education, marital status, household size, religion, whether belongs to minority ethnic group, employment status, and household income (quantiles). * *p* < 0.1; ** *p* < 0.05; *** *p* < 0.01.

### 5.4 Robustness checks

*Cohort Analysis*. To enable a more accurate comparison between migrants' self-reported data and information from their country of origin, we categorized cohorts according to year of birth and gender. Specifically, we define seven groups according to year of birth: 1988–2002, 1978–1987, 1968–1977, 1958–1967, 1948–1957, 1938–1947, and before 1938. For example, the self-reported health of first (second) generation female migrants born in 1985 is compared to the average self-reported health of women in their country of origin born between 1978 and 1987 (1958–1967). Table A9 in the Appendix shows that the results are very similar to those in Table 1, both with and without controls (columns 1–4). Controlling for the average self-assessed health of the country of residence for second-generation migrants reduces the size of the coefficients of interest, although it remains significant (Panel B, columns 5–6).

*Migrant Selection*. To test for potential selection into migration, we limit our analysis to migrants from EU countries with comparable rights and institutional development in both their country of origin and destination. Table 4 differentiates between samples of individuals born in EU countries and those who reside in EU countries but might be born elsewhere. This enables us to determine whether the effects are driven by migration from some of the non-EU countries represented in our sample. Again, we find large and significant coefficients across all regressions. When we examine the effect among migrants born in the EU, we still find evidence of cultural persistence across all generations.

**Table 4:** Cultural persistence of health status. Subsample of migrants within the European Union (EU).

	EU residents	(Parents) Born in the EU	EU residents and (parents) born in the EU
	(1)	(2)	(3)
Panel A. First generation migrants			
Self-assessed health at country of origin	0.593***	0.515***	0.316***
	[0.173]	[0.102]	[0.066]
	(0.061)	(0.076)	(0.082)
European regions fixed effects	Yes	Yes	Yes
Wave fixed effects	Yes	Yes	Yes
Controls	Yes	Yes	Yes
R^2^	0.28	0.22	0.21
Observations	16,419	9693	6683

Panel B. Second generation migrants			
Self-assessed health at country of origin	0.575***	0.425***	0.401***
	[0.180]	[0.087]	[0.084]
	(0.064)	(0.086)	(0.085)
European regions fixed effects	Yes	Yes	Yes
Wave fixed effects	Yes	Yes	Yes
Controls	Yes	Yes	Yes
R^2^	0.21	0.20	0.19
Observations	13,973	10,382	6791

Notes: The dependent variable is self-assessed health of first and second generation migrants who were born (or whose parents were born) and/or live in European Union countries (SAH=1 very good,…, SAH=5 very bad). OLS estimates, standardised coefficients are in brackets; standard errors (in parenthesis) are clustered at the country of origin level. Controls include age, education, marital status, household size, religion, whether belongs to minority ethnic group, employment status, and household income (quantiles). * *p* < 0.1; ** *p* < 0.05; *** *p* < 0.01.

We also consider migration selection using a two-step procedure. First, we use a probit model to estimate the likelihood of migration (Table A10 in the Appendix); the estimated parameters are then used to calculate the inverse Mills ratio, which is then added to the estimates that consider cohorts to link individuals' self-reported information and that of the country of origin (Table A9 in the Appendix). Our estimates suggest that the difference in the coefficient of country-of-origin self-reported health after including the Mills ratio from the coefficient calculated before is not statistically significant (95 %CI: −0.087, 0.028 for first-generation estimates, and 95 %CI: −0.102, 0.076 for second-generation estimates).

*Binarisanising self-assessed health*. Additionally, we investigate whether binarizing our variable—transforming self-reported health into a binary measure—affects our conclusions. Fig. A1 and Table A11 in the Appendix demonstrate that the results remain virtually unchanged.

*Genetic effects*. Arguably, common genetic factors could explain the similarities in health assessments of individuals from the same sending country. To assess cultural persistence in health assessments, we examine the cultural persistence of study self-assessed health while controlling for objective health measures. Estimates are consistent with baseline estimates. Our estimates are available across several specifications (details can be provided upon request).

*Other measures*. We also estimate the baseline specification for life satisfaction rather than self-reported health (Table 5). This provides additional evidence of the effect of other measures of self-assessed well-being. Table 5 suggests robust evidence of cultural transmission when such measures are employed (also see Fig. A2 in the Appendix).

**Table 5:** Cultural persistence of life satisfaction.

	First generation migrants			Second generation migrants	
	(1)	(2)	(3)	(4)	(5)
Life satisfaction at country of origin	0.440***	0.307***	0.290***	0.288**	0.281***
	[0.173]	[0.122]	[0.115]	[0.115]	[0.112]
	(0.103)	(0.064)	(0.061)	(0.118)	(0.056)
Citizen of country of residence			−0.093**		
			(0.040)		
Time in country of residence					
Within last year (reference)					
1 to 5 years			−0.033		
			(0.136)		
6 to 10 years			−0.166		
			(0.129)		
11 to 20 years			−0.266**		
			(0.124)		
More than 20 years			−0.298**		
			(0.141)		
Wave fixed effects	No	Yes	Yes	No	Yes
Controls	No	Yes	Yes	No	Yes
R^2^	0.03	0.11	0.12	0.01	0.13
Observations	27,431	24,829	24,410	24,295	22,239

Notes: The dependent variable is life satisfaction of first- and second-generation migrants who live in European countries. OLS estimates, standardised coefficients are in brackets; standard errors (in parenthesis) are clustered at the country of origin level. Specifications with controls (columns 2, 3, 5) include gender, age, education, marital status, household size, religion, whether belongs to minority ethnic group, employment status, and household income (quantiles). * *p* < 0.1; ** *p* < 0.05; *** *p* < 0.01.

In addition, we employ height and weight information collected in the seventh round of the ESS (in 2014) to define body-mass index (BMI), a more objective health measure, to run the baseline models. Age-standardized BMI averages per country for males and females were drawn from the NCD Risk Factor Collaboration. Table A12 in the Appendix first shows the results for self-reported health for round 7 of the ESS. The standardized coefficients of the variable of interest in the specifications with controls (columns 2–3) are only slightly lower than for the full sample (0.14 instead of 0.17). The results for BMI are also significant (columns 4–6), but the standardised coefficients are only half those for self-reported health, suggesting that the positive association for the subjective health measure does reflect, at least in part, the cultural persistence in how health is assessed, rather than the underlying health status. This is important given that the evidence suggests that the correlation between BMI and self-reported health is negligible. Finally, we consider potential differences in social norms. Specifically, add as a control the opinion on the statement “*men should have more right to a job than women when jobs are scarce*”, with response options: *agree, neither agree not disagree, disagree*, since it may also bear cultural information that may affect the health report in the destination country. We chose this variable because it is one of the few measuring attitudes that is available in both the ESS and the WVS. The results (not shown but available on request) are practically identical to those reported in Table 1, namely the (standardised) coefficients of the variable of interest in the models with controls remain significant and around 0.17.

## 6 Conclusion

This paper studies the hypothesis of cultural persistence in health self-assessments in a large and heterogeneous sample of Europeans. We have documented evidence of an association between migrants’ health assessments and that of their home countries (or that of their parents), which we argue capture what can be regarded as evidence of ‘cultural persistence’ in health assessments. This has been a question traditionally ignored in the evaluation of health programs across countries. Specifically, we document a clear association between subjective health assessments of first and second-generation immigrants (residing in 30 different European host countries and over 90 sending countries) and that of their home country.

Our findings suggest evidence that migrants' health assessments are associated with the average health status in their sending country, net of socio-demographic characteristics and other relevant controls. We report evidence that the correlation is stronger among older individuals and those residing in Northern Europe. We leverage on a large cross country variation which we beleive attenuates the likelihood of selection bias. We estimate that one standard deviation change in self-reported health in the sending country is associated with an increase in migrants' self-reported health of about 0.17 standard deviations.

Our interpretation of the results is that cultural reference points matter in making health assessments and are persistent across generations. Other explanations include some potential negative assimilation when health behaviors and cultural beliefs of the host country are perceived as advantageous, or the presence of selection bias in return migration which we cannot examine in our data as we cannot identify returning migrants. Finally, estimates are limited by any potentially unaccounted selection and the presence of genetic and epigenetic effects, alongside common migration wave-specific effects.

## CRediT authorship contribution statement

**Joan Costa-Font:** Conceptualization, Data curation, Formal analysis, Writing – original draft, Writing – review & editing. **Azusa Sato:** Conceptualization, Formal analysis. **Belen Saenz-de-Miera:** Conceptualization, Formal analysis, Validation, Visualization.

## Declaration of competing interest

This research has received no funding and we have no conflict of interest to disclose

## Appendix Supplementary materials

Image, application 1

## Footnotes

1 Wave 1 collected in 2002 was not considered, as this round does not include information on the country of origin of the migrants’ father and mother. Wave 9 collected in 2018 was also excluded, as the coding and country composition significantly changed. Some codes are now employed for groups of countries, which makes it impossible to identify the exact country of origin of migrants’ parents.

2 We specifically examine the effect when both parents are from the same country, but estimates are very similar (estimated provide upon request).
