# Supplementary material for: Cultural persistence of self-assessed health: A study of first- and second-generation migrants
Source: J Migr Health. 2024 Oct 28;11:100280. doi: 10.1016/j.jmh.2024.100280 (PMC11697404; doi:10.1016/j.jmh.2024.100280)
Supplement: Supplementary file 1 [file mmc1.docx]

**Appendix**

**Table A1. Sample description**

|  |  |  | **First-generation migrants** | | **Second-generation migrants** | |
| --- | --- | --- | --- | --- | --- | --- |
|  |  |  | n = 24,880 | | n=22,319 | |
| **Variable** | |  | Mean / proportion | s.e. | Mean / proportion | s.e. |
| Dependent variable | | |  |  |  |  |
|  | Self-assessed health of migrants | | 2.259 | 0.006 | 2.174 | 0.006 |
| Main independent variable | | |  |  |  |  |
|  | Self-assessed health in country of origin | | 2.280 | 0.002 | 2.278 | 0.002 |
| Sociodemographic controls | | |  |  |  |  |
|  | Female | | 0.556 | 0.003 | 0.540 | 0.003 |
|  | Age |  | 49.006 | 0.113 | 44.741 | 0.119 |
|  | Education | |  |  |  |  |
|  |  | Less than lower secondary | 0.103 | 0.002 | 0.065 | 0.002 |
|  |  | Lower secondary | 0.159 | 0.002 | 0.148 | 0.002 |
|  |  | Upper secondary | 0.324 | 0.003 | 0.410 | 0.003 |
|  |  | Post-secondary, non-tertiary | 0.106 | 0.002 | 0.109 | 0.002 |
|  |  | Tertiary | 0.304 | 0.003 | 0.266 | 0.003 |
|  |  | Other | 0.004 | 0.000 | 0.002 | 0.000 |
|  | Married | | 0.574 | 0.003 | 0.487 | 0.003 |
|  | Household size | | 2.760 | 0.009 | 2.911 | 0.010 |
|  | Religion | |  |  |  |  |
|  |  | None | 0.318 | 0.003 | 0.385 | 0.003 |
|  |  | Roman catholic | 0.214 | 0.003 | 0.196 | 0.003 |
|  |  | Protestant | 0.070 | 0.002 | 0.075 | 0.002 |
|  |  | Eastern orthodox | 0.142 | 0.002 | 0.110 | 0.002 |
|  |  | Islamic | 0.085 | 0.002 | 0.047 | 0.001 |
|  |  | Other | 0.172 | 0.002 | 0.188 | 0.003 |
|  | Belongs to ethnic minority | | 0.233 | 0.003 | 0.110 | 0.002 |
|  | Employment | |  |  |  |  |
|  |  | Paid work | 0.493 | 0.003 | 0.526 | 0.003 |
|  |  | Unemployed | 0.055 | 0.001 | 0.046 | 0.001 |
|  |  | Inactive | 0.452 | 0.003 | 0.428 | 0.003 |
|  | Quantiles of household income | |  |  |  |  |
|  |  | 1 | 0.164 | 0.002 | 0.124 | 0.002 |
|  |  | 2 | 0.183 | 0.002 | 0.156 | 0.002 |
|  |  | 3 | 0.167 | 0.002 | 0.160 | 0.002 |
|  |  | 4 | 0.129 | 0.002 | 0.153 | 0.002 |
|  |  | 5 | 0.120 | 0.002 | 0.144 | 0.002 |
|  |  | No response | 0.237 | 0.003 | 0.263 | 0.003 |
|  | Citizen of country of residence | | 0.611 | 0.003 |  |  |
|  | Time in country of residence | |  |  |  |  |
|  |  | Within last year | 0.009 | 0.001 |  |  |
|  |  | 1 to 5 years | 0.104 | 0.002 |  |  |
|  |  | 6 to 10 years | 0.110 | 0.002 |  |  |
|  |  | 11 to 20 years | 0.211 | 0.003 |  |  |
|  |  | More than 20 years | 0.567 | 0.003 |  |  |
| European Social Survey round | | |  |  |  |  |
|  | 2 |  | 0.121 | 0.002 | 0.119 | 0.002 |
|  | 3 |  | 0.112 | 0.002 | 0.114 | 0.002 |
|  | 4 |  | 0.158 | 0.002 | 0.149 | 0.002 |
|  | 5 |  | 0.158 | 0.002 | 0.149 | 0.002 |
|  | 6 |  | 0.165 | 0.002 | 0.171 | 0.003 |
|  | 7 |  | 0.146 | 0.002 | 0.150 | 0.002 |
|  | 8 |  | 0.142 | 0.002 | 0.149 | 0.002 |

Note: unweighted estimates; s.e. = standard error. SAH=1 very good,…, SAH=5 very bad.

**Table A2. Self-assessed health by sociodemographic characteristics and migration status**

|  |  |  | **First-generation migrants** | | **Second-generation migrants** | | **Natives** | |
| --- | --- | --- | --- | --- | --- | --- | --- | --- |
|  |  |  | n = 24,880 | | n=22,319 | | n =264,625 | |
| **Variable** | |  | Mean | s.e. | Mean | s.e. | Mean | s.e. |
| Self-assessed health | | | 2.259 | 0.006 | 2.174 | 0.006 | 2.250 | 0.002 |
| Sociodemographic controls | | |  |  |  |  |  |  |
|  | Female | | 2.325 | .009 | 2.231 | .009 | 2.317 | .003 |
|  | Age |  |  |  |  |  |  |  |
|  | 13-24 years | | 1.684 | .016 | 1.754 | .013 | 1.731 | .004 |
|  | 25-34 years | | 1.776 | .012 | 1.848 | .013 | 1.851 | .004 |
|  | 35-44 years | | 1.936 | .012 | 1.992 | .013 | 2.016 | .004 |
|  | 45-54 years | | 2.271 | .014 | 2.260 | .015 | 2.265 | .004 |
|  | 55-64 years | | 2.535 | .015 | 2.476 | .016 | 2.480 | .004 |
|  | 65+ years | | 2.890 | .013 | 2.759 | .016 | 2.789 | .004 |
|  | Education | |  |  |  |  |  |  |
|  |  | Less than lower secondary | 2.664 | .022 | 2.604 | .028 | 2.717 | .005 |
|  |  | Lower secondary | 2.384 | .016 | 2.244 | .017 | 2.327 | .004 |
|  |  | Upper secondary | 2.249 | .011 | 2.185 | .010 | 2.198 | .003 |
|  |  | Post-secondary, non-tertiary | 2.236 | .019 | 2.188 | .019 | 2.190 | .006 |
|  |  | Tertiary | 2.076 | .011 | 2.007 | .011 | 2.028 | .003 |
|  |  | Other | 2.198 | .091 | 2.000 | .152 | 2.249 | .046 |
|  | Married | | 2.242 | .008 | 2.191 | .009 | 2.277 | .002 |
|  | Religion | |  |  |  |  |  |  |
|  |  | None | 2.206 | .011 | 2.187 | .010 | 2.177 | .003 |
|  |  | Roman catholic | 2.166 | .013 | 2.260 | .014 | 2.327 | .003 |
|  |  | Protestant | 2.105 | .022 | 2.140 | .023 | 2.167 | .005 |
|  |  | Eastern orthodox | 2.598 | .017 | 2.603 | .018 | 2.456 | .006 |
|  |  | Islamic | 2.134 | .020 | 1.964 | .027 | 2.182 | .011 |
|  |  | Other | 2.316 | .017 | 1.870 | .015 | 2.071 | .013 |
|  | Belongs to ethnic minority | | 2.320 | .0130 | 2.279 | .019 | 2.348 | .010 |
|  | Employment | |  |  |  |  |  |  |
|  |  | Paid work | 1.975 | .007 | 1.999 | .008 | 2.006 | .002 |
|  |  | Unemployed | 2.135 | .024 | 2.158 | .028 | 2.172 | .009 |
|  |  | Inactive | 2.584 | .010 | 2.389 | .011 | 2.507 | .003 |
|  | Quantiles of household income | |  |  |  |  |  |  |
|  |  | 1 | 2.600 | .017 | 2.567 | .020 | 2.657 | .005 |
|  |  | 2 | 2.337 | .015 | 2.301 | .017 | 2.381 | .004 |
|  |  | 3 | 2.199 | .014 | 2.134 | .015 | 2.215 | .004 |
|  |  | 4 | 2.054 | .015 | 2.020 | .014 | 2.048 | .004 |
|  |  | 5 | 1.887 | .015 | 1.892 | .014 | 1.907 | .004 |
|  |  | No response | 2.304 | .013 | 2.180 | .012 | 2.260 | .004 |
| European Social Survey round | | |  |  |  |  |  |  |
|  | 2 |  | 2.245 | .018 | 2.226 | .018 | 2.217 | .005 |
|  | 3 |  | 2.341 | .018 | 2.265 | .018 | 2.312 | .005 |
|  | 4 |  | 2.276 | .016 | 2.157 | .016 | 2.277 | .004 |
|  | 5 |  | 2.234 | .016 | 2.209 | .017 | 2.285 | .005 |
|  | 6 |  | 2.259 | .015 | 2.154 | .015 | 2.250 | .004 |
|  | 7 |  | 2.252 | .016 | 2.112 | .016 | 2.181 | .005 |
|  | 8 |  | 2.221 | .017 | 2.127 | .016 | 2.204 | .005 |

Note: unweighted estimates; s.e. = standard error. SAH=1 very good,…, SAH=5 very bad.

**Table A3. Cultural persistence of health status. Baseline models with all the controls**

|  | **OLS** | | | **Oprobit** | | |
| --- | --- | --- | --- | --- | --- | --- |
|  | **First**  **generation** | | **Second generation** | **First**  **generation** | | **Second generation** |
| Self-assessed health at country of origin | 0.600*** | 0.584*** | 0.573*** | 0.774*** | 0.757*** | 0.766*** |
|  | (0.060) | (0.058) | (0.055) | (0.084) | (0.081) | (0.079) |
| Female | 0.047*** | 0.038*** | 0.058*** | 0.065*** | 0.053*** | 0.080*** |
|  | (0.012) | (0.011) | (0.014) | (0.015) | (0.015) | (0.019) |
| Age | 0.021*** | 0.017*** | 0.018*** | 0.027*** | 0.022*** | 0.024*** |
|  | (0.001) | (0.001) | (0.001) | (0.002) | (0.002) | (0.002) |
| Education (Reference =< Lower secondary) | | | | | | |
| Lower secondary | -0.088*** | -0.107*** | -0.011 | -0.096*** | -0.122*** | -0.001 |
|  | (0.026) | (0.025) | (0.046) | (0.034) | (0.033) | (0.057) |
| Upper secondary | -0.188*** | -0.208*** | -0.069 | -0.219*** | -0.246*** | -0.075 |
|  | (0.029) | (0.029) | (0.048) | (0.039) | (0.039) | (0.059) |
| Post-secondary, non-tertiary | -0.233*** | -0.253*** | -0.120** | -0.282*** | -0.310*** | -0.141** |
|  | (0.035) | (0.034) | (0.053) | (0.044) | (0.043) | (0.066) |
| Tertiary | -0.299*** | -0.298*** | -0.229*** | -0.372*** | -0.372*** | -0.291*** |
|  | (0.034) | (0.033) | (0.049) | (0.044) | (0.043) | (0.059) |
| Other | -0.148* | -0.146* | -0.078 | -0.158 | -0.156 | -0.110 |
|  | (0.079) | (0.076) | (0.119) | (0.103) | (0.099) | (0.168) |
| Married | -0.047*** | -0.038*** | -0.020 | -0.040** | -0.030* | -0.011 |
|  | (0.013) | (0.013) | (0.013) | (0.016) | (0.017) | (0.017) |
| Household size | 0.005 | -0.001 | -0.011* | 0.006 | -0.001 | -0.016** |
|  | (0.004) | (0.004) | (0.006) | (0.006) | (0.006) | (0.008) |
| Religion (Reference = None) | | | | | | |
| Roman catholic | -0.075*** | -0.065** | -0.034 | -0.099*** | -0.086*** | -0.049* |
|  | (0.025) | (0.025) | (0.023) | (0.032) | (0.033) | (0.030) |
| Protestant | -0.133*** | -0.125*** | -0.105*** | -0.182*** | -0.171*** | -0.143*** |
|  | (0.030) | (0.027) | (0.036) | (0.041) | (0.037) | (0.048) |
| Eastern orthodox | 0.071 | 0.076* | 0.109** | 0.067 | 0.075 | 0.123* |
|  | (0.049) | (0.044) | (0.054) | (0.067) | (0.061) | (0.071) |
| Islamic | 0.040 | 0.039 | -0.043 | 0.058 | 0.057 | -0.050 |
|  | (0.036) | (0.034) | (0.039) | (0.048) | (0.045) | (0.054) |
| Other | -0.100* | -0.103** | -0.269*** | -0.149** | -0.152** | -0.401*** |
|  | (0.053) | (0.050) | (0.036) | (0.065) | (0.063) | (0.052) |
| Belongs to minority ethnic group | -0.056*** | -0.071*** | -0.100*** | -0.070*** | -0.090*** | -0.132*** |
|  | (0.015) | (0.015) | (0.014) | (0.020) | (0.019) | (0.018) |
| Employment (Reference = Paid work) | | | | | | |
| Unemployed | 0.113*** | 0.122*** | 0.134*** | 0.154*** | 0.169*** | 0.181*** |
|  | (0.023) | (0.024) | (0.026) | (0.030) | (0.032) | (0.035) |
| Inactive | 0.215*** | 0.223*** | 0.156*** | 0.253*** | 0.266*** | 0.182*** |
|  | (0.015) | (0.016) | (0.016) | (0.020) | (0.022) | (0.018) |
| Quantiles of household income (Reference = Quantile 1) | | | | | | |
| Quantile 2 | -0.153*** | -0.159*** | -0.172*** | -0.188*** | -0.198*** | -0.213*** |
|  | (0.023) | (0.023) | (0.029) | (0.030) | (0.030) | (0.034) |
| Quantile 3 | -0.198*** | -0.212*** | -0.253*** | -0.240*** | -0.260*** | -0.310*** |
|  | (0.022) | (0.021) | (0.022) | (0.028) | (0.027) | (0.026) |
| Quantile 4 | -0.241*** | -0.260*** | -0.291*** | -0.297*** | -0.324*** | -0.360*** |
|  | (0.028) | (0.027) | (0.022) | (0.034) | (0.033) | (0.026) |
| Quantile 5 | -0.321*** | -0.342*** | -0.353*** | -0.414*** | -0.445*** | -0.447*** |
|  | (0.038) | (0.036) | (0.028) | (0.053) | (0.051) | (0.040) |
| No response | -0.211*** | -0.221*** | -0.227*** | -0.266*** | -0.281*** | -0.288*** |
|  | (0.028) | (0.027) | (0.034) | (0.032) | (0.031) | (0.039) |
| Citizen of country of residence |  | 0.029 |  |  | 0.038 |  |
|  |  | (0.025) |  |  | (0.032) |  |
| Time in country of residence (Reference = Within last year) | | | | | | |
| 1 to 5 years |  | 0.036 |  |  | 0.083 |  |
|  |  | (0.052) |  |  | (0.083) |  |
| 6 to 10 years |  | 0.092* |  |  | 0.167* |  |
|  |  | (0.055) |  |  | (0.086) |  |
| 11 to 20 years |  | 0.153** |  |  | 0.253*** |  |
|  |  | (0.063) |  |  | (0.096) |  |
| More than 20 years |  | 0.280*** |  |  | 0.417*** |  |
|  |  | (0.067) |  |  | (0.102) |  |
| Constant | 0.264 | 0.295* | 0.510*** |  |  |  |
|  | (0.160) | (0.159) | (0.144) |  |  |  |
| R^2^ / Pseudo R^2^ | 0.29 | 0.29 | 0.25 | 0.12 | 0.12 | 0.11 |
| Observations | 24,880 | 24,457 | 22,319 | 24,880 | 24,457 | 22,319 |

Notes: The dependent variable is self-assessed health of first- and second-generation migrants who live in European countries (SAH=1 very good,…, SAH=5 very bad). Standard errors (in parenthesis) are clustered at the country of origin level. * *p*<0.1; ** *p*<0.05; *** *p*<0.01.

**Table A4. Cultural persistence of health status. Baseline models with additional controls**

|  | | OLS | Oprobit | OLS | Oprobit | OLS | Oprobit |
| --- | --- | --- | --- | --- | --- | --- | --- |
|  | | (1) | (2) | (3) | (4) | (5) | (6) |
| **Panel A. First generation migrants** | |  |  |  |  |  |  |
| Self-assessed health at country of origin | | 0.561*** | 0.734*** | 0.205*** | 0.287*** | 0.386*** | 0.511*** |
|  |  | [0.159] | [0.030] | [0.058] | [0.012] | [0.109] | [0.021] |
|  |  | (0.060) | (0.083) | (0.047) | (0.065) | (0.055) | (0.073) |
| Citizen of country of residence | | 0.006 | 0.010 | -0.007 | -0.007 | -0.018 | -0.021 |
|  | | (0.025) | (0.032) | (0.015) | (0.020) | (0.025) | (0.032) |
| Time in country of residence | |  |  |  |  |  |  |
| Within last year (reference) | |  |  |  | | | |
| 1 to 5 years | | 0.042 | 0.089 | -0.010 | 0.015 | 0.038 | 0.086 |
|  | | (0.052) | (0.083) | (0.049) | (0.080) | (0.051) | (0.081) |
| 6 to 10 years | | 0.100* | 0.173** | 0.063 | 0.126 | 0.096* | 0.173** |
|  | | (0.055) | (0.086) | (0.051) | (0.083) | (0.053) | (0.085) |
| 11 to 20 years | | 0.158** | 0.256*** | 0.100* | 0.185** | 0.156** | 0.259*** |
|  | | (0.062) | (0.095) | (0.053) | (0.085) | (0.060) | (0.094) |
| More than 20 years | | 0.285*** | 0.422*** | 0.156*** | 0.260*** | 0.255*** | 0.389*** |
|  | | (0.067) | (0.102) | (0.057) | (0.089) | (0.065) | (0.099) |
| Ln(Per capita health expenditure) | |  |  |  |  | -0.264*** | -0.327*** |
|  | |  |  |  |  | (0.024) | (0.036) |
| R^2^ / Pseudo R^2^ | | 0.29 | 0.12 | 0.33 | 0.14 | 0.31 | 0.13 |
| Observations | | 24,394 | 24,394 | 24,394 | 24,394 | 24,338 | 24,338 |
|  | | | | | | | |
| **Panel B. Second generation migrants** | | | | | | | |
| Self-assessed health at country of origin | 0.544*** | | 0.735*** | 0.085 | 0.125* | 0.322*** | 0.446*** |
|  | [0.162] | | [0.023] | [0.025] | [0.004] | [0.096] | [0.014] |
|  | (0.056) | | (0.081) | (0.052) | (0.074) | (0.051) | (0.065) |
| Ln(Per capita health expenditure) |  | |  |  |  | -0.247*** | -0.315*** |
|  |  | |  |  |  | (0.018) | (0.029) |
| R^2^ / Pseudo R^2^ | 0.25 | | 0.11 | 0.29 | 0.13 | 0.26 | 0.11 |
| Observations | 22,276 | | 22,276 | 22,276 | 22,276 | 22,211 | 22,211 |
|  |  | |  |  |  |  |  |
| Wave fixed effects | Yes | | Yes | Yes | Yes | Yes | Yes |
| Controls | Yes | | Yes | Yes | Yes | Yes | Yes |
| European regions fixed effects | Yes | | Yes | No | No | No | No |
| Country of residence fixed effects | No | | No | Yes | Yes | No | No |

Notes: The dependent variable is self-assessed health of first- and second-generation migrants who live in European countries (SAH=1 very good,…, SAH=5 very bad). Standardised coefficients (OLS models) and average marginal effects on the probability of the worst self-assessed health (Oprobit models) are in brackets. Standard errors (in parenthesis) are clustered at the country of origin level. * *p*<0.1; ** *p*<0.05; *** *p*<0.01.

**Table A5. Cultural persistence of health status. Lag of average self-assessed health at country of origin**

|  | **First generation** | | **Second generation** | |
| --- | --- | --- | --- | --- |
|  | (1) | (2) | (3) | (4) |
| Self-assessed health at country of origin (1981-99) | 0.797*** | 0.521*** | 0.598*** | 0.483*** |
|  | [0.295] | [0.193] | [0.227] | [0.183] |
|  | (0.127) | (0.054) | (0.058) | (0.050) |
| Wave fixed effects | Yes | Yes | Yes | Yes |
| Controls | No | Yes | No | Yes |
| R^2^ | 0.09 | 0.31 | 0.05 | 0.25 |
| Observations | 20,079 | 20,079 | 18,363 | 18,363 |

Notes: The dependent variable is self-assessed health of first and second generation migrants who live in European countries (SAH=1 very good,…, SAH=5 very bad). OLS estimates (standardised coefficients in brackets); standard errors (in parenthesis) are clustered at the country of origin level. Specifications with controls (columns 2 and 4) include gender, age, education, marital status, household size, religion, whether belongs to minority ethnic group, employment status, and household income (quantiles). * *p*<0.1; ** *p*<0.05; *** *p*<0.01.

**Table A6. Cultural persistence of health status. Weighted estimates**

|  | OLS | | |  | Oprobit |  |
| --- | --- | --- | --- | --- | --- | --- |
|  | (1) | (2) | (3) | (4) | (5) | (6) |
| **Panel A. First generation migrants** |  |  |  |  |  |  |
| Self-assessed health at country of origin | 0.778*** | 0.552*** | 0.532*** | 0.917*** | 0.740*** | 0.717*** |
|  | [0.226] | [0.161] | [0.155] | [0.038] | [0.026] | [0.025] |
|  | (0.091) | (0.062) | (0.061) | (0.103) | (0.086) | (0.084) |
| Citizen of country of residence |  |  | 0.065* |  |  | 0.086* |
|  |  |  | (0.035) |  |  | (0.049) |
| Time in country of residence |  |  |  |  |  |  |
| Within last year (reference) |  |  |  |  |  |  |
| 1 to 5 years |  |  | 0.077 |  |  | 0.154 |
|  |  |  | (0.096) |  |  | (0.157) |
| 6 to 10 years |  |  | 0.171** |  |  | 0.290** |
|  |  |  | (0.083) |  |  | (0.137) |
| 11 to 20 years |  |  | 0.215** |  |  | 0.356** |
|  |  |  | (0.098) |  |  | (0.157) |
| More than 20 years |  |  | 0.336*** |  |  | 0.514*** |
|  |  |  | (0.099) |  |  | (0.158) |
| Wave fixed effects | Yes | Yes | Yes | Yes | Yes | Yes |
| Controls | No | Yes | Yes | No | Yes | Yes |
| R^2^ | 0.05 | 0.24 | 0.25 | 0.02 | 0.10 | 0.10 |
| Observations | 24,697 | 24,697 | 24,276 | 24,697 | 24,697 | 24,276 |
| **Panel B. Second generation migrants** | | | |  |  |  |
| Self-assessed health at country of origin | 0.772*** | 0.588*** |  | 0.906*** | 0.768*** |  |
|  | [0.240] | [0.183] |  | [0.036] | [0.027] |  |
|  | (0.096) | (0.058) |  | (0.119) | (0.080) |  |
| Wave fixed effects | Yes | Yes |  | Yes | Yes |  |
| Controls | No | Yes |  | No | Yes |  |
| R^2^ | 0.06 | 0.23 |  | 0.02 | 0.10 |  |
| Observations | 22,086 | 22,086 |  | 22,086 | 22,086 |  |

Notes: The dependent variable is self-assessed health of first and second generation migrants who live in European countries (SAH=1 very good,…, SAH=5 very bad). Standardised coefficients (OLS models) and average marginal effects on the probability of the worst self-assessed health (Oprobit models) are in brackets; standard errors (in parenthesis) are clustered at the country of origin level. Specifications with controls (columns 2-3, 5-6) include gender, age, education, marital status, household size, religion, whether belongs to minority ethnic group, employment status, and household income (quantiles). * *p*<0.1; ** *p*<0.05; *** *p*<0.01.

**Table A7. Cultural persistence of health status. Alternative definition of second-generation migrants that distinguishes paternal/maternal lineage**

|  | **Paternal lineage** | | | **Maternal lineage** | | |
| --- | --- | --- | --- | --- | --- | --- |
|  | (1) | (2) | (3) | (4) | (5) | (6) |
| Self-assessed health at country of origin | 0.760*** | 0.557*** | 0.321*** | 0.749*** | 0.543*** | 0.289*** |
|  | [0.224] | [0.164] | [0.095] | [0.219] | [0.159] | [0.085] |
|  | (0.095) | (0.058) | (0.057) | (0.090) | (0.063) | (0.057) |
| Ln(Per capita health expenditure) |  |  | -0.249*** |  |  | -0.255*** |
|  |  |  | (0.025) |  |  | (0.025) |
| Wave fixed effects | Yes | Yes | Yes | Yes | Yes | Yes |
| Controls | No | Yes | Yes | No | Yes | Yes |
| R^2^ | 0.05 | 0.25 | 0.26 | 0.05 | 0.25 | 0.26 |
| Observations | 15,604 | 15,604 | 15,542 | 14,751 | 14,751 | 14,680 |

Notes: The dependent variable is self-assessed health of second generation migrants who live in European countries (SAH=1 very good,…, SAH=5 very bad). OLS estimates (standardised coefficients in brackets); standard errors are clustered at the country of origin level. Specifications with controls (columns 2, 3, 5, 6) include gender, age, education, marital status, household size, religion, whether belongs to minority ethnic group, employment status, and household income (quantiles). * *p*<0.1; ** *p*<0.05; *** *p*<0.01.

**Table A8. Cultural persistence of health status. Heterogeneous effects by gender using alternative definition of second-generation migrants that distinguishes paternal/maternal lineage**

|  | | **OLS** | | **Oprobit** | |
| --- | --- | --- | --- | --- | --- |
|  | | **Female** | **Male** | **Female** | **Male** |
|  | | (1) | (2) | (3) | (4) |
| **Panel A. Second-generation migrants, paternal lineage** | | | |  |  |
| Self-assessed health at country of origin | | 0.555*** | 0.563*** | 0.730*** | 0.764*** |
|  |  | [0.164] | [0.166] | [0.021] | [0.024] |
|  |  | (0.036) | (0.040) | (0.109) | (0.077) |
| Wave fixed effects | | Yes | Yes | Yes | Yes |
| Controls | | Yes | Yes | Yes | Yes |
| R^2^ | | 0.26 | 0.23 | 0.11 | 0.10 |
| Observations | | 8,452 | 7,152 | 8,452 | 7,152 |
| **Panel B. Second-generation migrants, maternal lineage** | | | |  |  |
| Self-assessed health at country of origin | 0.577*** | | 0.497*** | 0.760*** | 0.692*** |
|  | [0.168] | | [0.147] | [0.027] | [0.018] |
|  | (0.038) | | (0.040) | (0.105) | (0.090) |
| Wave fixed effects | Yes | | Yes | Yes | Yes |
| Controls | Yes | | Yes | Yes | Yes |
| R^2^ / Pseudo R^2^ | 0.26 | | 0.23 | 0.11 | 0.10 |
| Observations | 7,921 | | 6,830 | 7,921 | 6,830 |

Notes: The dependent variable is self-assessed health of second-generation migrants who live in European countries (SAH=1 very good,…, SAH=5 very bad). Standardised coefficients (OLS models) and average marginal effects on the probability of the worst self-assessed health (Oprobit models) are in brackets; standard errors (in parenthesis) are clustered at the country of origin level. Controls include age, education, marital status, household size, religion, whether belongs to minority ethnic group, employment status, and household income (quantiles). * *p*<0.1; ** *p*<0.05; *** *p*<0.01.

**Table A9. Cultural persistence of health status. Specifications with cohorts based on year of birth and gender**

|  | OLS | Oprobit | OLS | Oprobit | OLS | Oprobit |
| --- | --- | --- | --- | --- | --- | --- |
|  | (1) | (2) | (3) | (4) | (5) | (6) |
| **Panel A. First generation migrants** |  |  |  |  |  |  |
| Self-assessed health at country of origin | 0.948*** | 1.150*** | 0.575*** | 0.731*** | 0.554*** | 0.706*** |
|  | [0.484] | [0.052] | [0.294] | [0.031] | [0.283] | [0.029] |
|  | (0.029) | (0.051) | (0.047) | (0.068) | (0.048) | (0.069) |
| Citizen of country of residence |  |  |  |  | 0.011 | 0.016 |
|  |  |  |  |  | (0.021) | (0.027) |
| Time in country of residence |  |  |  |  |  |  |
| Within last year (reference) |  |  |  | | | |
| 1 to 5 years |  |  |  |  | 0.034 | 0.082 |
|  |  |  |  |  | (0.053) | (0.083) |
| 6 to 10 years |  |  |  |  | 0.094* | 0.170** |
|  |  |  |  |  | (0.055) | (0.086) |
| 11 to 20 years |  |  |  |  | 0.153** | 0.255*** |
|  |  |  |  |  | (0.060) | (0.093) |
| More than 20 years |  |  |  |  | 0.276*** | 0.415*** |
|  |  |  |  |  | (0.065) | (0.099) |
| Observations | 24,841 | 24,841 | 24,841 | 24,841 | 24,418 | 24,418 |
| R^2^ / Pseudo R^2^ | 0.23 | 0.10 | 0.29 | 0.12 | 0.30 | 0.12 |
| **Panel B. Second generation migrants** | | | | | | |
| Self-assessed health at country of origin | 0.773*** | 0.953*** | 0.455*** | 0.602*** | 0.215*** | 0.305*** |
|  | [0.386] | [0.032] | [0.227] | [0.019] | [0.113] | [0.009] |
|  | (0.041) | (0.066) | (0.040) | (0.060) | (0.025) | (0.030) |
| Observations | 22,318 | 22,318 | 22,318 | 22,318 | 18,549 | 18,549 |
| R^2^ / Pseudo R^2^ | 0.15 | 0.06 | 0.25 | 0.11 | 0.27 | 0.12 |
| Self-assessed health at country of residence |  |  |  |  | Yes | Yes |
|  |  |  |  |  |  |  |
| Wave fixed effects | Yes | Yes | Yes | Yes | Yes | Yes |
| Controls | No | No | Yes | Yes | Yes | Yes |

Notes: The dependent variable is self-assessed health of first- and second-generation migrants who live in European countries (SAH=1 very good,…, SAH=5 very bad). First-generation migrants are compared with residents of their country of origin from the same cohort; second generation migrants are compared with residents of their parents’ country of origin from approximately the same cohort of their parents. Standardised coefficients (OLS models) and average marginal effects on the probability of the worst self-assessed health (Oprobit models) are in brackets; standard errors (in parenthesis) are clustered at the country-of-origin level. Specifications with controls (columns 3-6) include gender, age, education, marital status, household size, religion, whether belongs to minority ethnic group, employment status, and household income (quantiles). * *p*<0.1; ** *p*<0.05; *** *p*<0.01.

**Table A10. Probability of migrating**

|  | **First generation** | **Second generation** |
| --- | --- | --- |
| Self-assessed health at country of origin | 0.169*** | 2.100*** |
|  | (0.011) | (0.014) |
| Female | 0.025*** | -0.163*** |
|  | (0.007) | (0.008) |
| Age | -0.001*** | -0.046*** |
|  | (0.000) | (0.000) |
| Education (Reference =< Lower secondary) | | |
| Lower secondary | 0.102*** | -0.021 |
|  | (0.014) | (0.017) |
| Upper secondary | 0.088*** | 0.060*** |
|  | (0.013) | (0.015) |
| Post-secondary, non-tertiary | 0.219*** | 0.027 |
|  | (0.016) | (0.019) |
| Tertiary | 0.310*** | 0.006 |
|  | (0.014) | (0.017) |
| Other | 0.488*** | 0.180** |
|  | (0.067) | (0.091) |
| Married | 0.104*** | -0.062*** |
|  | (0.008) | (0.009) |
| Household size | -0.015*** | 0.000 |
|  | (0.003) | (0.003) |
| Religion (Reference = None) |  |  |
| Roman catholic | -0.058*** | -0.127*** |
|  | (0.009) | (0.010) |
| Protestant | -0.164*** | 0.070*** |
|  | (0.013) | (0.015) |
| Eastern orthodox | 0.150*** | -0.405*** |
|  | (0.012) | (0.014) |
| Islamic | 0.451*** | 0.094*** |
|  | (0.017) | (0.021) |
| Other | 1.027*** | 0.948*** |
|  | (0.013) | (0.015) |
| Belongs to minority ethnic group | -0.979*** | -0.055*** |
|  | (0.011) | (0.015) |
| Employment (Reference = Paid work) |  |  |
| Unemployed | 0.123*** | 0.058*** |
|  | (0.017) | (0.020) |
| Inactive | -0.032*** | -0.031*** |
|  | (0.008) | (0.009) |
| Quantiles of household income (Reference = Quantile 1) | | |
| Quantile 2 | 0.003 | 0.083*** |
|  | (0.013) | (0.015) |
| Quantile 3 | -0.013 | 0.215*** |
|  | (0.013) | (0.015) |
| Quantile 4 | -0.101*** | 0.282*** |
|  | (0.014) | (0.016) |
| Quantile 5 | -0.095*** | 0.383*** |
|  | (0.015) | (0.017) |
| No response | -0.099*** | 0.030** |
|  | (0.012) | (0.014) |
| Constant | -1.024*** | -4.358*** |
|  | (0.030) | (0.037) |
| Wave fixed effects | Yes | Yes |
| Observations | 309,380 | 309,380 |

Notes: The dependent variable indicates whether the individual is a first (second) generation migrant (Yes=1). Probit estimates; standard errors are clustered at the country of origin level. * *p*<0.1; ** *p*<0.05; *** *p*<0.01.

**Figure A1. Cultural persistence of health status. Correlation of self-assessed health (SAH) between European residents of first- and second-generation migrants and country of origin. Binary specification of self-assessed health**

1. First-generation migrants


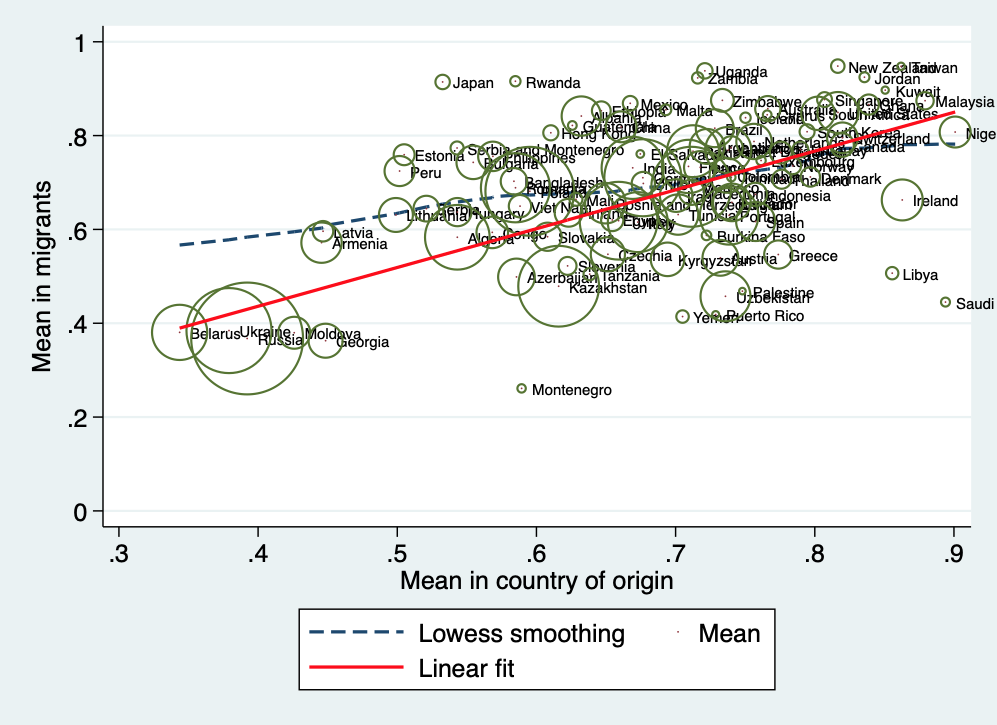


1. Second-generation migrants


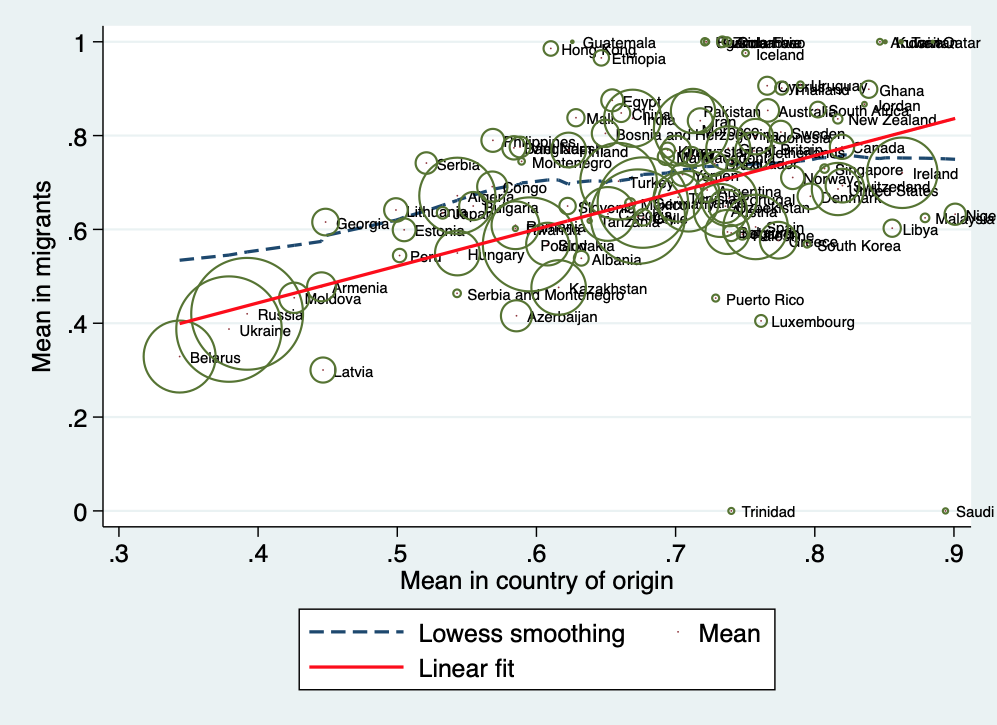


Note: The size of the green circles represents the number of migrants from each country.

**Table A11. Cultural persistence of health status. Binary specification of self-assessed health**

|  | **First generation migrants** | | | **Second generation migrants** | |
| --- | --- | --- | --- | --- | --- |
| SAH at country of origin | 0.940*** | 0.722*** | 0.702*** | 0.722*** | 0.632*** |
|  | (0.141) | (0.066) | (0.062) | (0.053) | (0.048) |
| Female |  | -0.039*** | -0.033*** |  | -0.034*** |
|  |  | (0.007) | (0.008) |  | (0.010) |
| Age |  | -0.011*** | -0.008*** |  | -0.009*** |
|  |  | (0.001) | (0.001) |  | (0.001) |
| Education (reference =< lower secondary) | | | | | |
| Lower secondary |  | 0.014 | 0.026 |  | -0.009 |
|  |  | (0.020) | (0.019) |  | (0.025) |
| Upper secondary |  | 0.073*** | 0.085*** |  | 0.020 |
|  |  | (0.019) | (0.018) |  | (0.023) |
| Post-secondary, non-tertiary |  | 0.073*** | 0.083*** |  | 0.041 |
|  |  | (0.023) | (0.022) |  | (0.026) |
| Tertiary |  | 0.121*** | 0.120*** |  | 0.106*** |
|  |  | (0.023) | (0.021) |  | (0.020) |
| Other |  | 0.070 | 0.072 |  | -0.006 |
|  |  | (0.051) | (0.046) |  | (0.074) |
| Married |  | 0.019** | 0.013 |  | 0.004 |
|  |  | (0.008) | (0.008) |  | (0.008) |
| Household size |  | -0.006** | -0.003 |  | 0.006** |
|  |  | (0.003) | (0.003) |  | (0.003) |
| Religion (reference = none) | | | | | |
| Roman catholic |  | 0.038*** | 0.031** |  | 0.016 |
|  |  | (0.013) | (0.013) |  | (0.013) |
| Protestant |  | 0.075*** | 0.067*** |  | 0.052*** |
|  |  | (0.019) | (0.017) |  | (0.018) |
| Eastern orthodox |  | -0.060** | -0.064*** |  | -0.055** |
|  |  | (0.026) | (0.022) |  | (0.025) |
| Islamic |  | -0.007 | -0.006 |  | 0.025 |
|  |  | (0.021) | (0.019) |  | (0.017) |
| Other |  | 0.040 | 0.040 |  | 0.092*** |
|  |  | (0.026) | (0.025) |  | (0.018) |
| Belongs to minority ethnic group |  | 0.040*** | 0.049*** |  | 0.042*** |
|  |  | (0.009) | (0.008) |  | (0.009) |
| Employment (reference = paid work) | | | | | |
| Unemployed |  | -0.061*** | -0.070*** |  | -0.083*** |
|  |  | (0.018) | (0.018) |  | (0.017) |
| Inactive |  | -0.102*** | -0.110*** |  | -0.075*** |
|  |  | (0.009) | (0.009) |  | (0.010) |
| Quantiles of household income (reference = quantile 1) | | | | | |
| Quantile 2 |  | 0.063*** | 0.067*** |  | 0.081*** |
|  |  | (0.012) | (0.012) |  | (0.015) |
| Quantile 3 |  | 0.088*** | 0.097*** |  | 0.115*** |
|  |  | (0.012) | (0.012) |  | (0.010) |
| Quantile 4 |  | 0.116*** | 0.129*** |  | 0.140*** |
|  |  | (0.015) | (0.014) |  | (0.010) |
| Quantile 5 |  | 0.161*** | 0.174*** |  | 0.180*** |
|  |  | (0.021) | (0.019) |  | (0.013) |
| No response |  | 0.089*** | 0.097*** |  | 0.095*** |
|  |  | (0.017) | (0.015) |  | (0.014) |
| Citizen of country of residence |  |  | -0.013 |  |  |
|  |  |  | (0.014) |  |  |
| Time in country of residence (reference = within last year) | | | | | |
| 1 to 5 years |  |  | 0.001 |  |  |
|  |  |  | (0.051) |  |  |
| 6 to 10 years |  |  | -0.057 |  |  |
|  |  |  | (0.054) |  |  |
| 11 to 20 years |  |  | -0.108* |  |  |
|  |  |  | (0.056) |  |  |
| More than 20 years |  |  | -0.188*** |  |  |
|  |  |  | (0.055) |  |  |
| Observations | 27,517 | 24,880 | 24,457 | 24,398 | 22,319 |

Notes: The dependent variable is self-assessed health of first- and second-generation migrants who live in European countries (SAH=1 good or very good). Probit estimates (marginal effects are reported); standard errors (in parenthesis) are clustered at the country of origin level. * *p*<0.1; ** *p*<0.05; *** *p*<0.01.

**Figure A2. Cultural persistence of life satisfaction. Correlation of life satisfaction between European residents of first- and second-generation migrants and country of origin**

1. First-generation migrants


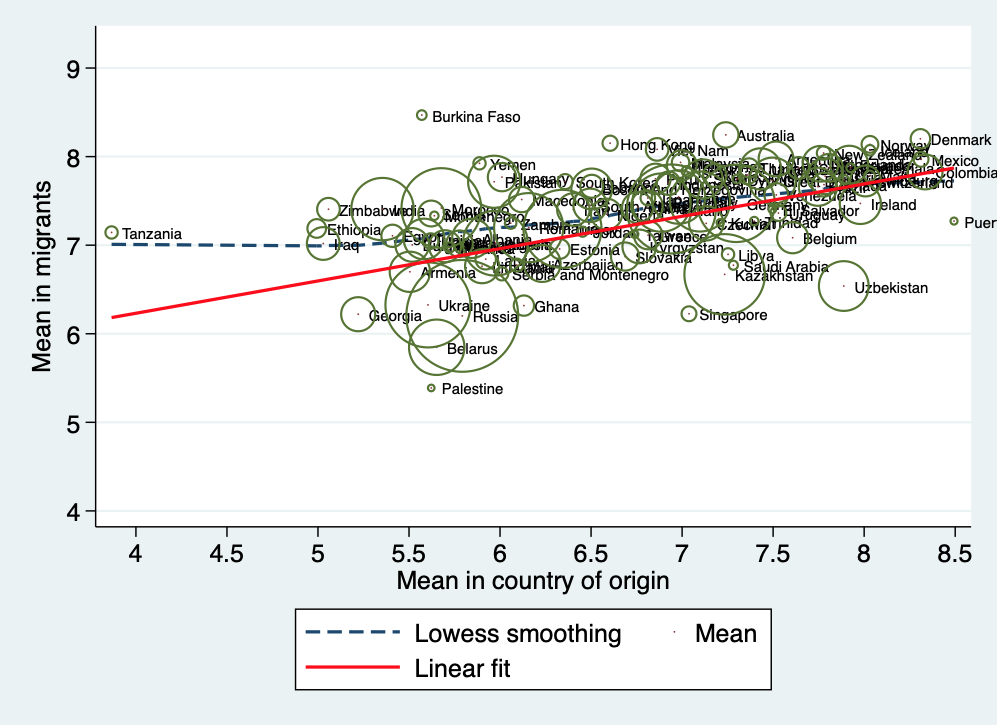


1. Second-generation migrants


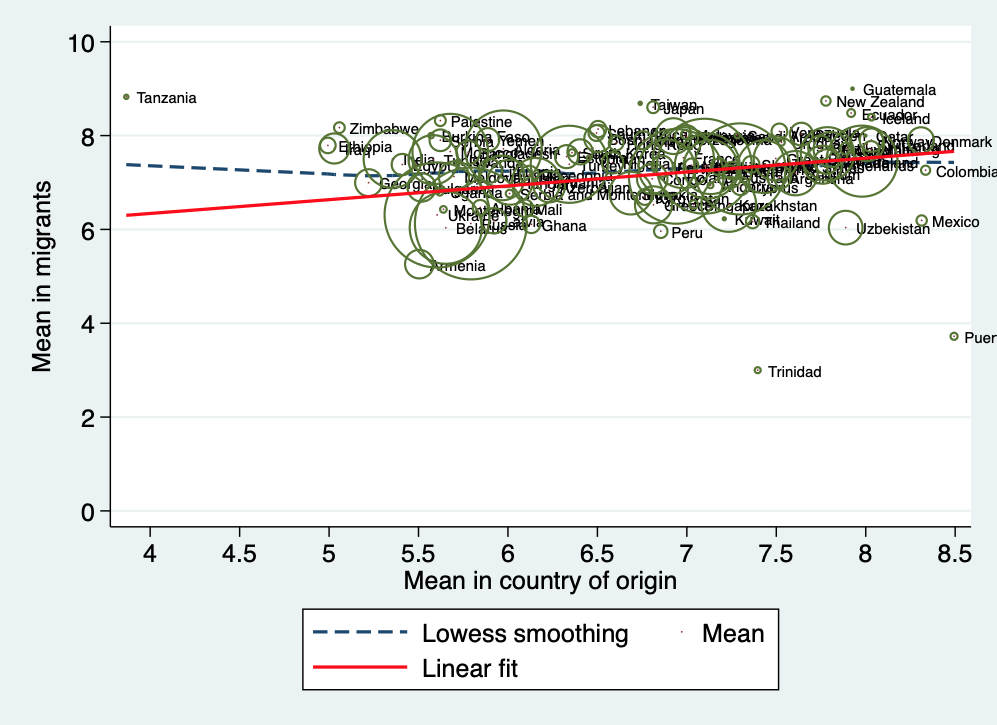


Note: The size of the green circles represents the number of migrants from each country.

**Table A12. Cultural persistance of health. Subjective (self-assessed health) vs. physcal measure (body-mass index)**

|  | Self-assessed health | | | Body-mass index | | |
| --- | --- | --- | --- | --- | --- | --- |
|  | (1) | (2) | (3) | (4) | (5) | (6) |
| **Panel A. First generation migrants** |  |  |  |  |  |  |
| Health at country of origin | 0.647*** | 0.460*** | 0.469*** | 0.316*** | 0.243*** | 0.247*** |
|  | [0.198] | [0.141] | [0.143] | [0.092] | [0.071] | [0.070] |
|  | (0.131) | (0.060) | (0.059) | (0.088) | (0.068) | (0.059) |
| Citizen of country of residence |  |  | -0.057 |  |  | 0.238 |
|  |  |  | (0.039) |  |  | (0.223) |
| Time in country of residence |  |  |  |  |  |  |
| Within last year (reference) |  |  |  | | | |
| 1 to 5 years |  |  | -0.106 |  |  | -0.866 |
|  |  |  | (0.224) |  |  | (0.671) |
| 6 to 10 years |  |  | -0.036 |  |  | -0.278 |
|  |  |  | (0.212) |  |  | (0.762) |
| 11 to 20 years |  |  | 0.032 |  |  | -0.518 |
|  |  |  | (0.225) |  |  | (0.721) |
| More than 20 years |  |  | 0.157 |  |  | -0.454 |
|  |  |  | (0.227) |  |  | (0.763) |
| Observations | 3,549 | 3,549 | 3,511 | 3,549 | 3,549 | 3,511 |
| R^2^ | 0.04 | 0.27 | 0.28 | 0.01 | 0.10 | 0.10 |
| Controls | No | Yes | Yes | No | Yes | Yes |

Notes: The dependent variables are self-assessed health and body-mass index of first- and second-generation migrants who live in European countries (SAH=1 very good,…, SAH=5 very bad). OLS estimates; standardised coefficients are in brackets. Standard errors (in parenthesis) are clustered at the country-of-origin level. Specifications with controls (columns 2-3, 5-6) include gender, age, education, marital status, household size, religion, whether belongs to minority ethnic group, employment status, and household income (quantiles). * *p*<0.1; ** *p*<0.05; *** *p*<0.01
